# Serological and molecular analysis of parvovirus B19 infection in Mayan women with systemic lupus erythematosus in Mexico

**DOI:** 10.25100/cm.v48i3.2981

**Published:** 2017-09-30

**Authors:** Guillermo Valencia Pacheco, Yumi E Nakazawa Ueji, Edwin A Rodríguez Dzul, Angélica V Angulo Ramírez, Ricardo F López Villanueva, Irma G Quintal Ortiz, Elsy P Rosado Paredes

**Affiliations:** 1 Laboratorio de Hematología, Centro de Investigaciones Regionales Dr. Hideyo Noguchi, Universidad Autónoma de Yucatán. Mérida, Yucatán, México; 2 Hospital General Agustín O'Horán. Mérida, Yucatán, México; 3 Servicios de Salud de Yucatán, Hospital General Regional . Mérida, Yucatán, México

**Keywords:** Autoimmunity, environmental factors, autoantibodies, Systemic Lupus Erythematosus, parvovirus B19, Parvoviridae Infections, Mayan population, Mexico, Autoinmunidad, factores ambintales, autoanticuerpos, Lupus eritematoso sistémico, parvovirus B19, infección por parvoviridae, población Maya, Mexico

## Abstract

**Background:**

Systemic lupus erythematosus (SLE) is a systemic autoimmune disease that mainly affects women, characterized by the production of autoantibodies. Its causal agent is unknown, but the combination of environmental, hormonal and genetic factors may favor the development of the disease. Parvovirus B19 has been associated with the development of SLE, since it induces the production of anti-single stranded DNA antibodies. It is unknown whether PV-B19 infection is an environmental factor that trigger or reactivate SLE in the Mexican Mayan population.

**Aim:**

A preliminary serological and molecular study of PV-B19 infection in Mayan women with established SLE was done.

**Methods:**

IgG and IgM anti PV-B19 were evaluated in 66 SLE patients and 66 control subjects, all women of Mayan origin. Viral DNA and viral load were analyzed by qPCR.

**Results:**

Insignificant levels of IgM were observed in 14.3% (4/28) of the patients and 11.4% (4/35) of control subjects. IgG was detected in 82.1% (23/28) of the patients and 82.9% (29/35) of control subjects, but were significantly higher in patients. Viral DNA was found in 86.0% (57/66) of the patients and 81.0% (54/66) of control subjects. Viral load, quantified in 28/66 patients and 31/66 controls which were positive for IgM and IgG, was significantly higher in controls.

**Conclusion:**

The high prevalence of PV-B19 in Yucatan, and the presence of IgM, IgG, and viral load in Mayan women with established SLE suggest that PV-B19 infection could be an environmental factor to trigger or reactivate SLE.

## Resumen

**Antecedentes:**

Lupus eritematoso sistémico (LES) es una enfermedad sistemica autoinmune que afecta principalmente a las mujeres, caracterizada por la producción de autoanticuerpos. El agente causaal es desconocido. Pero la combinación de factores ambientales, hormonales y genéticos podría favorecer el desarrollo de la enfermedad. El parvovirus B19 se asoció con el desarrollo de LES, debido a que induce la producción de anticuerpos anti-cadena simple de DNA. Es desconocido si la infección PV-B19 es un factor ambiental que desencadena o reactiva LES en la población mexicana Maya.

**Objetivo:**

Se realizó un estudio serológico y molecular preliminar de la infección de PV-B19 en mujeres Mayas con LES.

**Métodos:**

Se evaluó IgG and IgM anti PV-B19 en 66 pacientes con LES y 66 controles sanos, todas las mujeres fueron de origen Maya. DNAViral y la carga viral fueron analizadas por qPCR.

**Resultados:**

Se determinaron niveles insignificantes de IgM en el 14.3% (4/28) de las pacientes y en el 11.4% (4/35) de los controles. IgG se detectó en el 82.1% (23/28) de los pacients y en el 82.9% (29/35) de los controles. Hubo un alta significancia en los pacientes con LES. DNA viral se encontró en el 86.0% (57/66) de los pacientes y en el 81.0% (54/66) de los controles. La carga viral se cuantifico en 28/66 pacientes y en 31/66 de los controles, la cual fueron positivos para IgM e IgG; fue significativamente mas alta en los controles.

**Conclusión:**

La alta prevalencia de PV-B19 en Yucatan y la presencia de IgM, IgG y una carga viral en mujeres Mayas con LES sugiere que la infección con PV-B19 poria ser un factor ambiental que desencadene o reactive el LES

## Introduction 

Systemic lupus erythematosus (SLE) is a chronic inflammatory systemic autoimmune disease of unknown etiology, caused by the interaction of genetic and environmental factors that contribute to the production of autoantibodies against self-antigens. The disease has a worldwide distribution and predominantly affects women ^1^ ^-^ ^3^. Asian countries such as China, Hong Kong, Philippines and Japan, has reported more cases, and others like the United States, France, Spain, UK, and some regions of Australia, has presented increase in patients ^4^ ^,^ ^5^. Several studies have been conducted in patients from different populations (Asian, European, American), but few in Mexican population. 

Mexico has an admixed Mestizo population with a genetic pool from the Amerindian and the Spanish ^6^. The ancestry data derived from the HapMap project, which included Mexicans, shows that the Yucatan mestizos are the only ethnic group with Amerindian ancestry that are geographically distant from other Amerindian groups ^7^. On the other hand, Mexican individuals with SLE appear to have a more severe disease than European, a lower age of onset and a higher frequency of disease activity flares. Also, it has been reported that the prevalence of SLE in Yucatan (0.7%) is slightly higher than the national prevalence (0.6%) ^8^ ^,^ ^9^.

Environmental factors such as bacterial, parasitic, fungal and viral infections have been associated with the pathogenesis of the disease in genetically predisposed patients ^3^ ^,^ ^10^. It has been reported that various viruses and bacteria can produce superantigens which, through mechanisms such as adjuvant effect (bystander) and molecular mimicry, induce activation of autoreactive T and B lymphocytes. Viral particles in infected B lymphocytes can lead to the production of autoantibodies and cytokines such as IFN-α, contributing to autoimmune and inflammatory mechanism. Epstein-Barr virus (EBV), Cytomegalovirus (CMV), Human T-lymphotropic virus 1 (HTLV-1), and Parvovirus B19 (PV-B19) have been linked to the pathogenesis of SLE ^11^ ^,^ ^12^.

Human PV-B19, which was identified in 1975 by Yvonne Cossart and her colleagues ^13^, is a small single-strand DNA virus (22-24 nm diameter) that causes a variety of diseases in humans. Its icosahedral capsid is composed of two identical structural proteins, VP1 (83 kDa) and VP2 (58 kDa), except for an additional fragment of 277 amino acids at the amino terminus of VP1. This unique VP1 region is external to the capsid, with many linear epitopes and phospholipase A2 activity (PLA2), which causes cytotoxicity and infectivity. PV-B19 also has the nonstructural protein NS1 (77kDa) involved in its transcription and translation ^14^. Three genotypic variants of PV-B19 have been identified: genotype 1 has a worldwide distribution; genotype 2 has been detected in patients from several European countries, the United States and Brazil; genotype 3 is most frequently in Africa and less frequently in other geographical areas ^15^ ^-^ ^22^.

PV-B19 is transmitted by respiratory aerosol spread from individuals acutely infected, or by parenteral transmission via blood transfusion and blood products ^23^ ^-^ ^25^. The virus replicates in the erythroblasts in the bone marrow, which express the blood group P antigen or globoside (Gb4), the alpha5beta1 integrin, and the Ku80 protein ^26^. Viral replication, leading to viremia on day 6, appears to be important in most clinical manifestations. Most infections are asymptomatic or have mild clinical pictures, but when the infection is associated with age-influenced clinical disorders or immune and hematologic status, it presents a wide variety of clinical manifestations that may be confused with systemic autoimmune diseases such as rheumatoid arthritis (RA), progressive systemic sclerosis, Sjögren's syndrome (SS), vasculitis or SLE ^27^ ^-^ ^29^. PV-B19 infection may be misdiagnosed as a new onset SLE, but at the same time, can both occur simultaneously in some patients.

PV-B19 is a ubiquitous virus, distributed worldwide, which can infect any age group. Primary infection usually occurs in childhood and adolescence. Seroprevalence (presence of specific IgG denoting past infection) increases with age. In industrialized countries, it is estimated from 2% to 10% in children under five years, that could increase up to 50% at the age of 15 and in adults it varies from 40% to 70%. By the age of 70, it becomes 80% to 100% ^30^ ^,^ ^31^. Japan and Germany have reported high infections rates in pregnant women ^32^ ^,^ ^33^.

In Mexico, there are few clinical and epidemiological studies of PV-B19. Tapia *et al.* ^34^, determined IgM and IgG in 128 people from groups considered at high risk of infection with PV-B19, and healthy people of all age groups and both sexes, in the Infectious Diseases Hospital at la Raza Medical Center. The results showed the presence of infection especially in women (63.2%) of 25-44 years (48.4%), with exanthems, habitual abortion, and anemia in immunocompromised patients or hematologic disorders. In 61 patients (47.6%), higher IgG antibodies were found, and only 4 of them had IgM. Vera *et al.* ^35^,conducted a preliminary prospective study of 102 pregnant women in two rural towns of Yucatan, Mexico, founding a seroprevalence of 5.9% of IgM and 11.8% of IgG; confirming the presence of PV-B19 infection in these populations.

Several studies have been focused on the diagnosis of PV-B19 infection, but the relationship of PV-B19 with stablished SLE has not been studied in Mayan population of Mexico. Our objective was to perform a preliminary serological and molecular analysis of PV-B19 infection in women of the Mayan population with established SLE and healthy women. IgM and IgG anti-PVB19, presence of viral DNA and viral load were evaluated in both groups.

## Materials and Methods

### SLE patients

 Sixty-six SLE women of Mayan origin, were recruited at the Rheumatology outpatient of the Agustin O'Horán and ISSSTE Regional Hospital, Yucatan. Diagnosis was established according American College of Rheumatology (ACR) criteria ^36^, and disease activity was evaluated by SLEDAI score ^37^. SLE women reported having different times with the disease. Sixty-six healthy women of the same origin with no history of autoimmune or infectious diseases as controls were studied. None of them were receiving any treatment. All women included gave their informed consent, according to the Declaration of Helsinki. The study was approved by the Research Ethics Committee of the Agustin O'Horán Hospital of Yucatan (CIE-008-1-11). All women gave 10 mL of venous peripheral blood (without anticoagulant) in one collection to obtain serum. 

### IgM and IgG anti-PV-B19

Two commercially available ELISA kits for detection of anti-B19 IgM (EIA-3504) and anti-B19 IgG (EIA-3503) (DRG Instruments GmbH, Germany) were used ^38^. Microtiter wells as a solid phase are coated with recombinant Parvovirus B19 antigen (VP1 proteins). Diluted serum from patients and controls, and ready-for-use controls, are pipetted into these wells. During incubation Parvovirus B19-specific antibodies of positive serum and controls are bound to the immobilized antigen. Subsequently, the specific human anti-IgG or anti-IgM conjugated to horseradish peroxidase (HRP) is added. The reaction is visualized by adding tetramethylbenzidine (TMB) which generates a blue color. The enzymatic reaction is stopped by addition of sulfuric acid solution (H_2_SO_4_), which develops a yellow color. The color intensity is proportional to antibody concentration. Reading was performed at a wavelength of 450 nm in an ELISA reader (model BioTek(r) ELx800), and antibody concentration was determined by the following formula:

***Antibody concentration= (Abs) (10)/ CO***

Where:

Abs = sample absorbance

10 = constant to compare absorbance (cut-off and control samples)

CO = Cut-off control mean absorbance

Antibody concentration is expressed in DU (DRG units, exclusive measure of supplier used to have a parameter measurement of immunoglobulins), taking the absorbance cutoff control as reference. Each assay was performed in duplicate using the positive, negative and cutting controls, contained in the kit. Results were interpreted as follows: IgM positive >11 DU, IgG positive >12 DU, IgM negative <9 DU and, IgG negative <8.5 DU, respectively.

### DNA isolation 

DNA extraction was performed on IgM and IgG positive sera from patients and controls by saturated phenol method ^39^. This procedure was based on the classical phenol/cloroform extraction method using 200 (L of serum samples. Solution of chloroform-isoamyl alcohol (24:1) was added to separate protein, and the DNA was precipitated with 100% ethanol and 7.5 M ammonium acetate for 24 h at -20° C. The DNA pellet was washed twice with 70% ethanol, dried in the oven at 37° C for 1 h, and then resuspended in 30 (L of ultrapure water. After incubation of 20 min at 56° C, the DNA was quantified in a spectrophotometer (Nanodrop (tm) Thermo Scientific® 2000c), at wavelengths of 260 and 280 nm. DNA was stored at -20° C prior to use. 

### Cloning 

To determine the presence of viral DNA and to quantify the viral load, a segment of the NS1 protein was cloned from the viral DNA extracted from the serum of a patient diagnosed with PVB19 and was used as a positive control. The 168 bp band corresponding to the protein NS1, was amplified using the Maxima Hot Start Taq DNA polymerase (Thermo Scientific) and primers (0.5 µM) as follows: 4 min initial denaturation at 95º C, following by 40 cycles of denaturation (95° C for 30 sec), primer annealing (55° C, 30 sec), extension step of 1 min at 72º C, and a final holding stage at 72° C for 10 min. PCR products were identified by electrophoresis in agarose 1% stained with GelRed (GelRed(tm) Nucleic Acid Gel Stain. Biotium). The band was purified by centrifugation (Wizard SV Gel and PCR Clean-up system kit, Promega), and ligated to pCR 2.1-TOPO cloning vector (TOPO-TA Cloning kit, Invitrogen by Life Technologies). The ligation product was introduced into chemically competent E. coli cells (One Shot® TOP10 Competent Cells, Invitrogen by Life Technologies) at 42° C/30 s, and were seeded in LB solid medium with kanamycin (50 µg/mL) overnight at 37° C. White colonies expressing plasmid were selected and seeded in the liquid LB medium overnight at 37° C. Plasmid was purified (PureLink Quick Plasmid DNA Miniprep kit. Invitrogen by Life Technologies), and visualized by electrophoresis in agarose 1% stained with GelRed. The presence of the insert was determined by PCR using the Maxima Hot Start Taq DNA polymerase (Thermo Scientific). Purified plasmids were analyzed by BLAST and compared with the reported sequences of NS1 gene of PV-B19 in the GenBank. 

### Detection of PV-B19 DNA

Viral DNA (15 ng) was amplified by real time-PCR, designed according to MIQE guidelines ^40^. Primers and probes used in amplification reactions were described by Bonvicini *et al*. ^41^, (B19 primer forward 5'-CGCCTGGAACASTGAAACCC-3', B19 primer reverse 5´-TCAACCCCWACTAACAGTTC-3', and genotype 1 probe 6FAMGTTGTAGCTGCATCGTGGGAAGAMGBNFQ). They were designed by Applied Biosystems and directed against the non-structural protein 1 (NS1, 616-2631 nucleotides) of PV-B19 genotype 1. Amplification conditions were as follows: 20 sec of initial denaturation at 95º C, following by 50 cycles of amplification: denaturation (95° C for 3 s), primer annealing (55° C, 40 s), and a final incubation 30 s at 60º C. Amplification was carried out in the StepOne (tm) Real-Time PCR System equipment using TaqMan Fast Virus 1 step Master Mix (Applied Biosystems). Analysis of viral DNA (presence/absence of NS1, and genotype 1) was performed according to the CT (cycle threshold). The CT is the cycle at which the fluorescence level reaches a certain amount (the threshold). This method directly uses the CT information generated to calculate relative expression in target and reference samples, using as reference a negative sample ^42^.

### Viral load

IgM and IgG positive samples were quantified by real time PCR using the same primers and probe, described above. Viral load was quantified using a standard curve assay with different copy number of the plasmid containing the fragment of 168 bp of the NS1 gene (4,099bp). The number of copies of the 6-point standard curve was determined using the URI Genomics & Sequencing Center software (http://cels.uri.edu/gsc/cndna.html). This calculation was based on the assumption that the average weight of a base pair (bp) is 650 Daltons. This means that one mole of a bp weighs 650 g and that the molecular weight of any double stranded DNA template can be estimated by taking the product of its length (in bp) and 650. The inverse of the molecular weight is the number of moles of template present in one gram of material. Using Avogadro's number, 6.022x10^23^ molecules/mole, the number of molecules of the template per gram is calculated. The number of copies of template was estimated by multiplying by 1x10^9^ to convert to ng and then multiplying by the amount of template (in ng). The formula used, starting from an initial concentration of 1ng, was: 

(1 ng x 6.022 x 10^23)^ / (4099 x 1 x 10^9^ x 650) = 2.25 x 10^8^ copies.

Five serial dilutions at 10 were included (Table 1). Each sample and standard curve were tested by triplicate. Amplification conditions were as follows: 2 min pre-heating at 50º C, 10 min polymerase activation at 95º C, following by 50 cycles of denaturation (95° C for 15 s), primer annealing (55° C, 40 s), extension step of 20 s at 72º C, and a final holding stage at 60° C for 30 s. Amplification reaction was performed in the StepOne (tm) Real-Time PCR System equipment (Applied Biosystems), using the Maxima Probe/ROX master mix (Thermo Scientific), primers (0.5 µM), and probe (0.1 µM). The number of copies in the samples was calculated with the StepOne software taking into account the average of CT values obtained with respect to the standard curve. Viral load are expressed in copies per milliliter of serum (cps/mL).

**Table 1:** Number of copies of the standard curve, determined in triplicate with the URI Genomics & Sequencing Center software, as described in Material and methods.

Standard curve (ng)	Number of copies
1	2.25 x 10^8^
0.1	2.25 x 10^7^
0.01	2.25 x 10^6^
0.001	2.25 x 10^5^
0.0001	2.25 x 10^4^
0.00001	2.25 x 10^3^

### Statistical Analysis 

Wilcoxon matched-pairs signed rank test was used to assess the significance of any difference in values of IgM and IgG, and viral load (cps/mL) among SLE patients and control subjects (*p* <0.05). Correlation analysis was done using the Pearson correlation coefficient. In all comparisons, the level of significance was p <0.05, using the Graph Pad Prism 5 software. 

## Results

### Characteristics of SLE patients and controls 

The average age of the patients and controls was 39.03 and 38.18 years, respectively. The average time with disease in patients was 9 years (Table 2). All female patients were under treatment, 56.1% of them had active disease determined by SLEDAI (>4).

**Table 2:** Characteristics of SLE patients (n=66).

Features	SLE patients
Mean age (year)	39.0
Mean Disease duration (year)	9.9
SLEDAI (%)	
Active (≥ 4)	56.1
Nonactive (< 4)	43.9
Locality in the Yucatan State(%)	
Mérida	53.0
Hunucmá	3.0
Maxcanú	3.0
Motul	3.0
Peto	4.6
Progreso	3.0
Other in Yucatan State	30.0
Treatment(%)	
Prednisone	54.5*
Azathioprine	39.4*
Metotrexate	13.6*
Deflazacort	18.2*
Hydroxycloroquine	19.7*

SLEDAI: Systemic Lupus Erythematosus Disease Activity Index.

* Percentage of patients receiving the drug in combination with other one

### Levels of IgM and IgG anti PV-B19 

**Figure 1:**
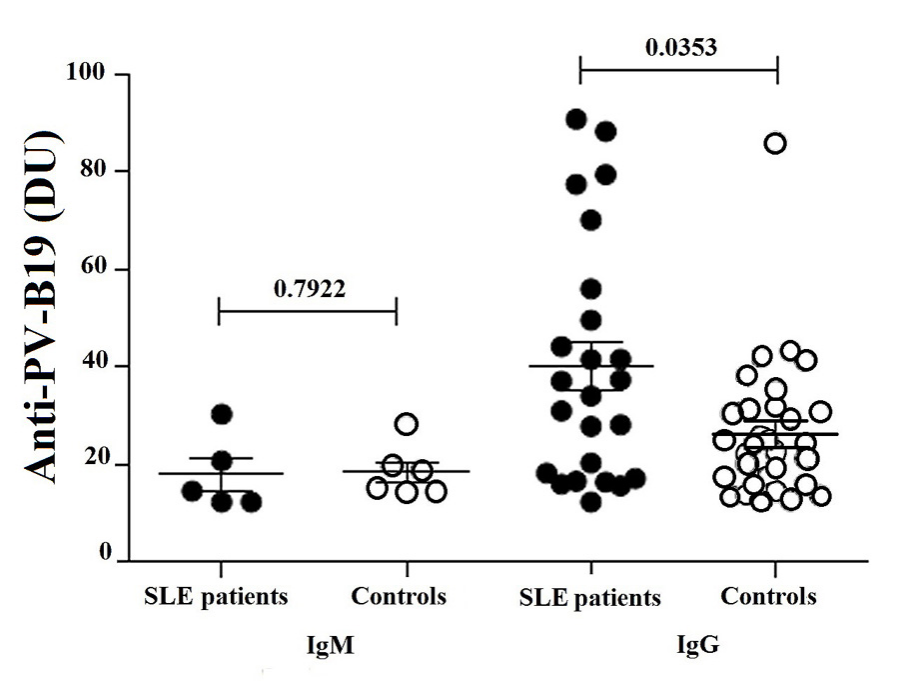
Levels of IgM and IgG anti-PV-B19 in SLE patients (n= 28) and controls (n= 30), analyzed by ELISA as described in Material and methods. Results expressed in DU units are presented in scatter plots and mean with SME (standard mean error). Wilcoxon matched-pairs signed rank test was used to assess the difference of expression among SLE patients and control subject (*p* <0.05).

**Figure 2:**
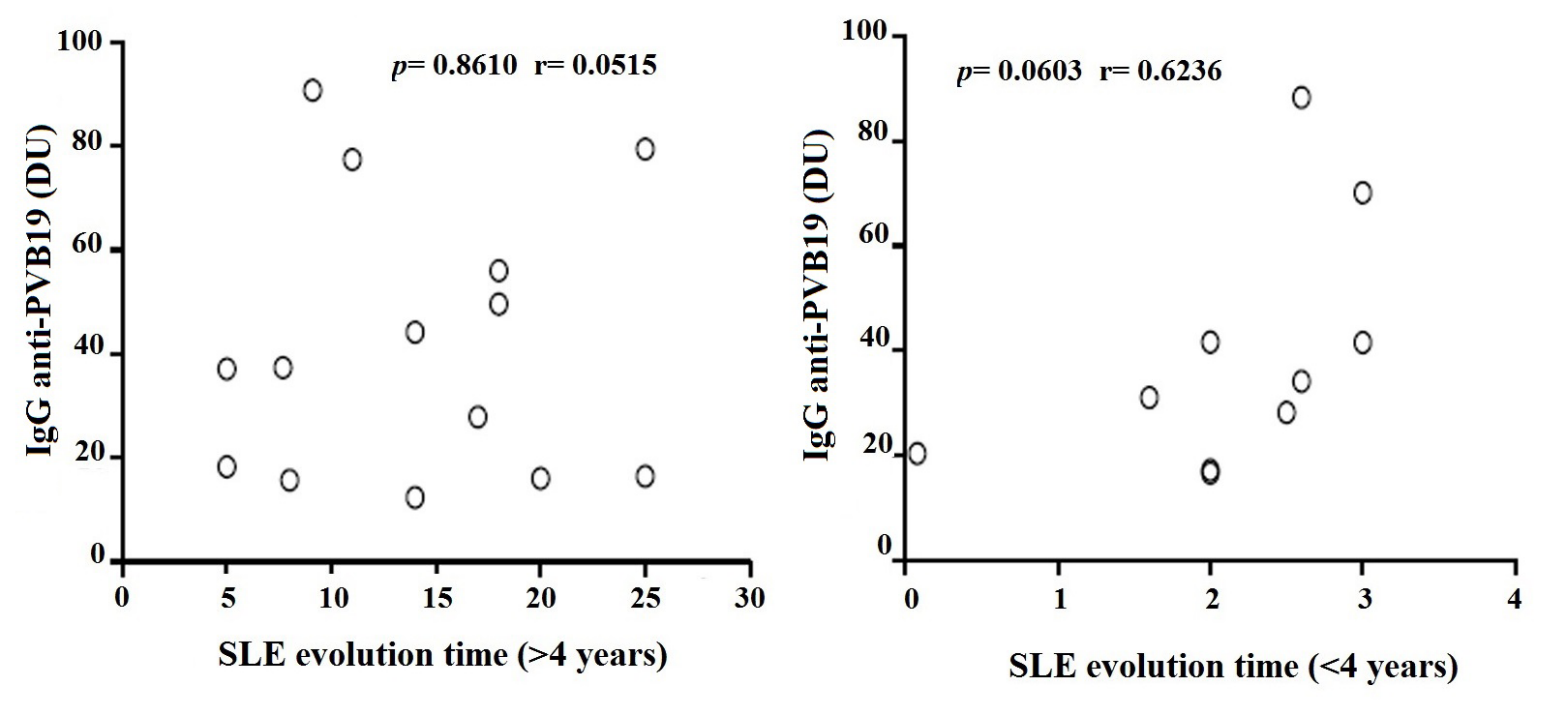
Correlation analysis of IgG anti-PV-B19 with evolution time (≥4 or ≤4 years) in SLE patients. Results are presented in scatter plots. Pearson correlation test was used to assess correlation. r= Pearson correlation coefficient; *p* <0.05.

**Figure 3:**
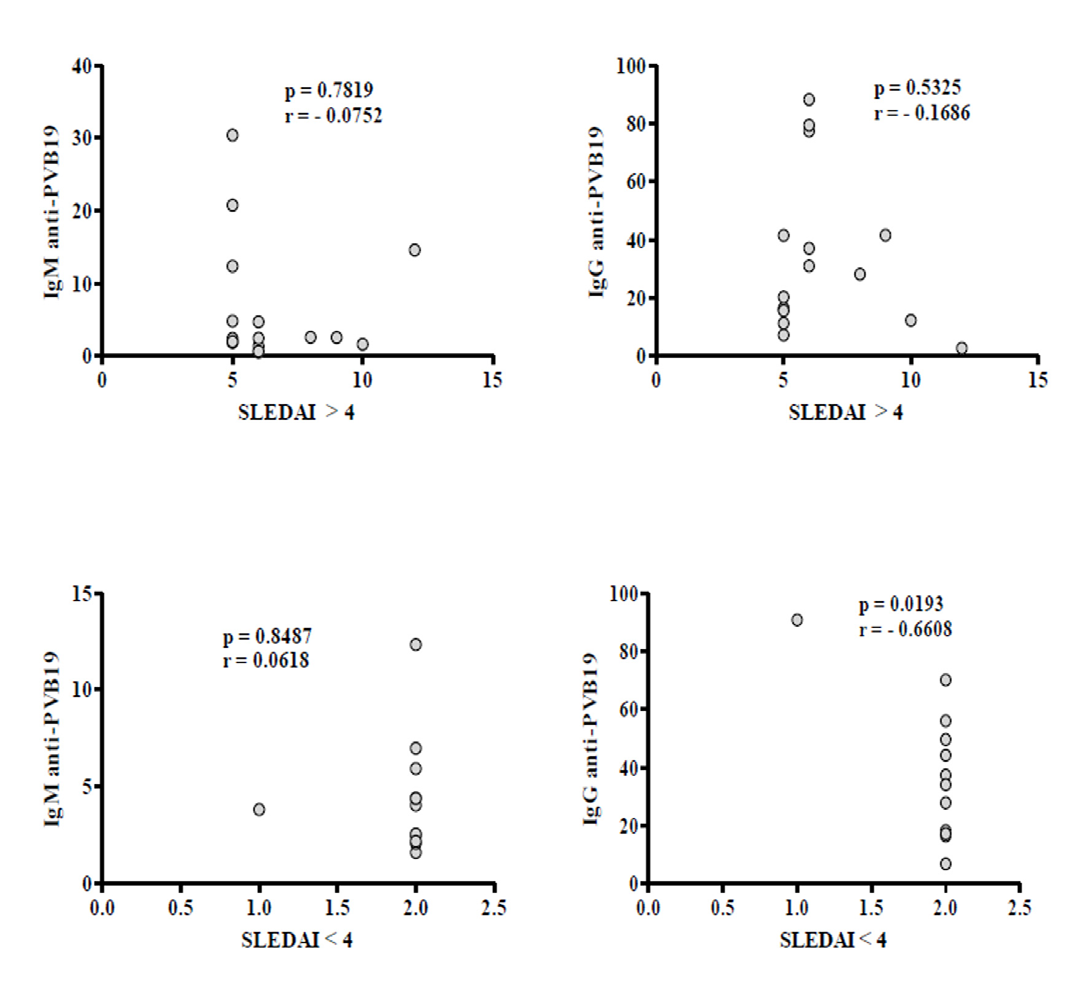
Correlation analysis of IgM and IgG anti-PV-B19 with disease activity (SLEDAI >4 or <4) in SLE patients. Results are presented in scatter plots. Pearson correlation test was used to assess correlation. r= Pearson correlation coefficient; *p* <0.05.

Antibodies were detected in 42.4% (28/66) of SLE patients and 53.0% (35/66) of controls. We found 14.3% (4/28) of patients and 11.4% (4/35) of controls with no significant levels of IgM (*p*= 0.7922). On the other hand, 82.1% (23/28) of patients and 82.9% (29/35) of controls showed IgG, but significantly higher levels were detected in patients (*p*= 0.0353) (Table 3 and Fig. 1). Only one patient and two controls have both IgG and IgM. We found 58.3% (14/24) of patients with disease duration of 4 years or more presented IgG, but no correlation was observed. Correlation was also not observed in those who presented less than 4 years (41.7%, 10/24) (Fig. 2). Association analysis of IgG and IgM with SLEDAI was performed but no correlation was observed between IgM levels and disease activity (SLEDAI >4). However, IgG levels showed significant negative correlation in patients with lower disease activity (SLEDAI <4) (Fig. 3).

**Table 3:** IgM and IgG anti-PV-B19 in SLE patients (n = 66) and controls (n = 66), analyzed by ELISA as described in Material and methods.

Antibody	SLE patients (n (%))	Controls (n (%))
IgM >11 DU	4 (14.3)	4 (11.4)
IgG >12 DU	23 (82.1)	29 (82.9)
IgG and IgM	1 (3.6)	2 (5.7)
Total	28 (42.4)	35 (53.0)

### Detection of PV-B19 DNA and viral load 

**Figure 4:**
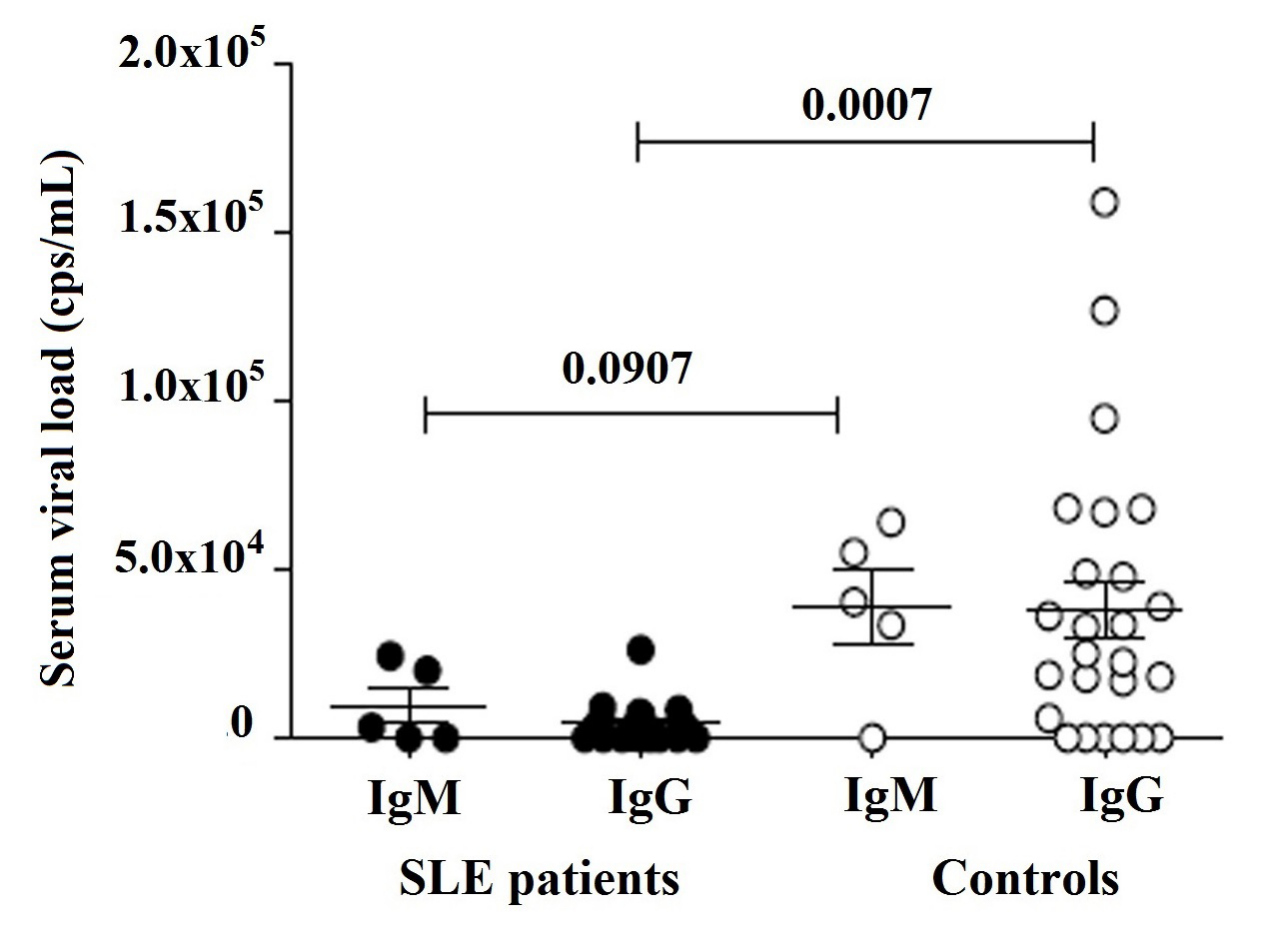
Viral load in serum of SLE patients (28/66) and controls (31/66) with IgM or IgG, analyzed by qPCR as described in Material and methods. Results expressed in copies/mL are presented in scatter plots and mean with SME (standard mean error). Wilcoxon matched-pairs signed rank test was used to assess the difference of expression among SLE patients and control subject (*p* <0.05).

**Figure 5:**
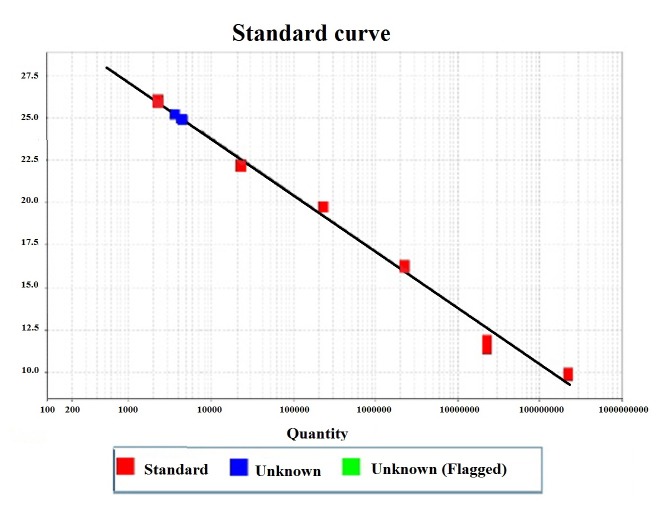
Graph representing the number of copies of the standard curve with the CT values of an analyzed sample, as described in Material and methods. The red symbols indicate the points of the standard curve and the blue ones of a sample, taken from the StepOne software.

**Figure S1:**
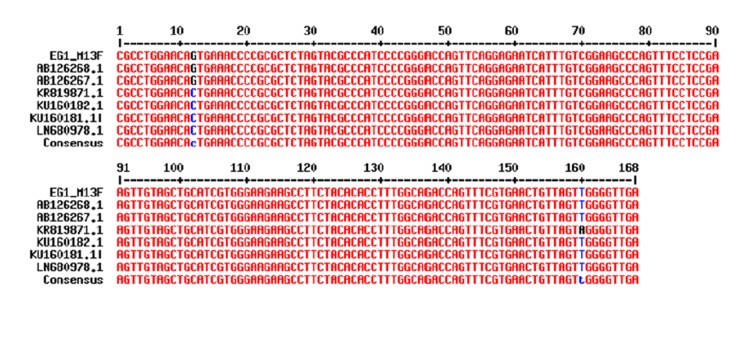
Alignment of sequenced fragment of NS1 protein of PV-B19 with reported sequences in GenBank.

The sequenced fragment of NS1 protein, used as positive control, was analyzed by BLAST and showed 100% of homology with reported sequences of NS1 gene of PV-B19 in GenBank 43-45 (Fig. S1). The sequence was registered in the GenBank (BankIt1994458 Human KY680313). PV-B19 DNA of genotype 1 was detected in 86.4% (57/66) and 81.8% (54/66) of patients and controls, respectively (Table S1). Viral load was quantified in 28/66 SLE patients and 30/66 healthy controls, which were positive for IgM and IgG (Table S2). We found that 67.9% (19/28) of patients presented viral load: 10.7% (3/28) with IgM and 57.1 % (16/28) with IgG. It was also found viral load in 80.0% (24/30) of the controls: 13.3% (4/30) with IgM and 66.7 % (20/30) with IgG, respectively (Table 4). Viral load was no detected in patients with IgM (1/28), IgG (7/28), or both (1/28), neither in controls subjects with IgG (5/30), or IgM and IgG (1/30) (Table S2). No correlation of IgM or IgG with viral load was found in both groups; however, viral load was significantly higher in the controls with IgG (Fig. 4). A graph representing the number of copies of the standard curve is shown with the CT values of a sample (Fig. 5).

**Table 4:** Serum viral load (cps/mL) in SLE patients (n = 28) and controls (n = 30) with IgM or IgG, analyzed by qPCR and ELISA, respectively, as described in Material and methods.

Viral load/antibodies	SLE patients (%)	Healthy controls (%)
cps/mL (+) IgM (+)	3/28 (10.7)	4/30 (13.3)
cps/mL (+) IgG (+)	16/28 (57.1)	20/30 (66.7)
cps/mL (-) IgM (+)	1/28 (3.6)	--
cps/mL (-) IgG (+)	7/28 (25.0)	5/30 (16.7)
cps/mL (-) IgM/IgG (+)	1/28 (3.6)	1/30 (3.3)

## Discussion

During viral infection the humoral immune response is crucial to limit infection. In immunocompetent individuals, viremia begins 6 days after infection and decreases days later with the presence of antibodies against VP1 and VP2 proteins. In acute infection IgM antibodies are detectable in the first 3 days of the infection and undetectable between 60 and 90 days, but it could remain elevated between 3 and 6 months ^46^ ^-^ ^48^. Some authors report that acute PV-B19 infection may trigger its onset or exacerbate preexisting SLE ^49^ ^,^ ^50^. 

This is the first report on PV-B19 infection in women with stablished SLE of the Mayan population in Mexico. IgM antibodies against VP1 were detected in patients with confirmed SLE who showed disease activity (SLEDAI >5), and had an average of 9.8 years with the disease. Although IgM levels in the patients were not different from the controls, and showed no correlation with the disease activity, probably due to the duration of the disease and the treatment, data suggest recent infection in our patients and seems to correlate with the reactivation of the disease. This supports that PV-B19 infection is associated with confirmed SLE as etiopathogenic factor, and it corresponds to what was suggested by Ramos *et al* ^51^.

IgG is detected days after IgM, indicating resolution of infection and past or chronic infection, providing lifelong immunity ^47^ ^,^ ^48^. In our study, high levels of IgG antibodies were detected in SLE patients, supporting past or chronic infection. Unlike the data reported by Pugliese *et al*. ^52^, who found a significant correlation between IgG anti-PV-B19 and SLE, we found no correlation between IgG and SLEDAI; the inverse correlation observed between IgG with low disease activity (SLEDAI <4), is probably due to high IgG values in a single patient, which we consider not representative. Our data suggest that IgG levels appear to increase in SLE patients likely due to support therapy for chronic PV-B19 infection, since all of our patients were being treated with anti-inflammatory drugs, corticosteroids and immunosuppressive agents. In this regard, it has been described that the use of corticosteroids, immunosuppressive agents, and biological therapies may increase the risk of viral infection in SLE patients and PV-B19 infection could become chronic or severe on them ^53^ ^,^ ^54^. However, longitudinal studies are required to confirm this.

PV-B19 genotype 1 was detected in SLE patients and controls, and viral load was quantified in those patients and controls with high levels of IgM or IgG antibodies. We found no correlation between IgM or IgG antibodies and viral load in both groups; however, higher viral load was found in controls confirming presence of PV-B19 in the region, and supporting the prevalence of infection in the Mayan population. IgG antibodies and viral load in patients seem to support the chronic infection associated with immunosuppression by therapy. Viral load was no detected in some patients with IgM, IgG or both. In this regard, it has been reported that viremia disappears at day 10 post infection, whereas IgM (10-12 days) and IgG (14 days) start to synthesize. In this stage, viral particles are not detected, indicating that the reason for not detecting viral DNA in some of our patients and controls with high titer of IgG and/or IgM, could be that those individuals were in day 12 of infection, when virus is not present. However, longitudinal studies are required to confirm this.

In our control women, no clinical symptoms suggestive recent infection or illness were observed, but IgM and IgG antibodies, as well as presence of DNA and viral load were detected. Despite the differences in sample size and populations studied, the data support the IgM and IgG seroprevalence found by Vera *et al*. ^35^, and confirm the circulation of the virus in the Mayan population. Viral load was no detected in some controls with IgG or IgM/IgG antibodies. In this regard, it has been reported that immunocompetent individuals produce antibodies that effectively eliminate viremia within a few days of infection, and the infection is often not developed, is asymptomatic or has mild clinical manifestations (like the common cold) ^55^, which seems to agree with what we found in our controls. On the other hand, there have also been rare cases of chronic PV-B19 infection in healthy individuals, with involvement of the central nervous system, causing nonspecific symptoms such as fatigue, fever, arthralgia and myalgia, which may hinder the diagnostic ^56^. None of our controls women manifest some of these symptoms, however, longitudinal studies are needed to evaluate the association of PV-B19 infection with neurological, autoimmune or hematological disorders in the immunocompetent Mayan population.

## Conclusion

The high prevalence of PV-B19 in Yucatan, and the presence of IgM, IgG, and viral load in Mayan women with established SLE suggest that PV-B19 infection could be an environmental factor to trigger or reactivate SLE. However, longitudinal studies and a large sample are required to confirm the association of PV-B19 with the development of SLE, as well as the effect of immunosuppressive therapy on the resurgence of the virus.

## Annexes

**Table S1:** Presence/absence of viral DNA and IgM or IgG in SLE patients (n = 66) and

SLE Patients	Presence Absence	IgM (DU)	IgG (DU)	Healthy controls	Presence Absence	IgM (DU)	IgG (DU)
1	P	2.50	3.74	1	A	2.40	2.20
2	P	1.90	16.00	2	P	2.80	1.76
3	P	3.83	90.75	3	A	2.00	4.80
4	P	5.95	56.00	4	P	2.80	1.40
5	P	4.05	1.83	5	P	3.70	4.10
6	P	4.42	16.48	6	P	6.70	1.70
7	A	5.20	3.00	7	P	2.60	24.20
8	P	2.46	16.63	8	P	3.30	7.60
9	P	2.57	34.12	9	A	1.40	6.10
10	P	6.99	70.16	10	P	3.11	35.30
11	P	4.75	88.25	11	P	2.00	2.10
12	P	1.62	17.24	12	P	2.28	14.54
13	A	2.10	3.90	13	P	5.70	2.00
14	P	1.46	2.82	14	P	1.82	7.49
15	A	0.00	1.53	15	P	2.78	7.46
16	A	1.44	2.83	16	P	4.24	1.39
17	A	1.38	9.98	17	P	4.20	2.59
18	P	7.13	2.88	18	P	1.64	1.16
19	P	2.19	3.37	19	A	2.00	4.20
20	P	1.66	12.36	20	P	3.69	41.26
21	P	2.60	41.59	21	P	2.23	86.02
22	A	4.10	1.70	22	A	2.04	2.71
23	P	1.40	2.00	23	P	3.13	24.94
24	P	1.40	31.00	24	P	3.64	13.72
25	P	4.10	2.50	25	P	2.06	22.42
26	P	7.10	1.10	26	P	2.48	4.21
27	A	5.38	3.10	27	P	2.15	13.65
28	P	2.00	1.40	28	A	6.90	6.60
29	P	1.24	7.29	29	P	1.80	17.44
30	P	1.11	2.40	30	A	1.70	3.08
31	P	5.15	2.73	31	P	2.18	2.84
32	P	5.86	2.76	32	P	1.67	13.85
33	P	5.08	2.29	33	P	2.50	7.50
34	P	2.81	3.41	34	P	2.23	2.26
35	P	0.60	2.00	35	P	1.53	30.76
36	P	0.66	0.79	36	A	2.65	1.95
37	P	5.72	1.20	37	P	4.79	1.25
38	P	3.73	1.75	38	P	10.90	16.00
39	P	0.56	77.46	39	P	5.39	25.00
40	P	0.69	79.46	40	P	3.70	2.80
41	P	5.12	8.31	41	P	1.51	1.60
42	P	4.85	20.34	42	A	1.76	0.00
43	P	6.62	6.98	43	A	5.10	2.60
44	P	30.41	11.37	44	P	5.97	31.52
45	P	20.77	15.73	45	P	4.16	22.18
46	P	8.75	11.15	46	P	2.70	5.40
47	P	12.34	6.88	47	P	14.42	42.10
48	P	5.77	10.20	48	P	2.78	19.44
49	P	2.01	41.53	49	P	7.34	31.77
50	P	4.32	7.35	50	P	4.15	29.55
51	P	2.43	7.37	51	P	1.80	8.27
52	A	11.02	11.34	52	P	4.15	11.43
53	A	1.96	3.35	53	P	3.65	12.73
54	P	4.16	9.51	54	P	2.92	20.17
55	P	4.89	4.65	55	P	9.37	43.27
56	P	1.73	7.91	56	-	3.10	11.81
57	P	2.61	28.23	57	P	6.28	21.28
58	P	1.37	4.00	58	P	18.84	11.54
59	P	2.62	0.00	59	P	10.49	24.23
60	P	1.61	5.13	60	P	9.73	38.18
61	P	2.52	10.54	61	P	11.52	30.42
62	P	2.08	9.18	62	P	14.41	16.08
63	P	2.1.	49.66	63	P	28.21	1.78
64	P	2.19	44.17	64	P	1.52	3.52
65	P	2.47	3.71	65	P	1.97	2.16
66	P	4.41	27.85	66	-	8.19	9.43

Presence: P

Absence: A

**Table S2:** Serum viral load (cps/mL) in SLE patients and controls with IgM or IgG, analyzed by qPCR and ELISA, respectively, as described in Material and methods.

SLE Patients	cps/mL	IgM (DU)	IgG (DU)	Healthy controls	cps/mL	IgM (DU)	IgG (DU)
1	4201.636791	25	3.74	1	159046.3715	28	1.76
2	4117.944717	19	1.60	2	68096.33636	26	2.42
3	0	383	90.75	3	0	311	3.53
4	9168.199539	595	5.60	4	122609.1675	228	14.54
5	0	405	1.83	5	5914.156437	42	2.59
6	0	442	16.48	6	67065.8493	223	86.02
7	26209.05876	246	16.63	7	16777.81105	313	24.94
8	0	257	34.12	8	94853.63007	364	13.72
9	8431.143761	699	70.16	9	39084.53369	206	22.42
10	2591.694593	475	88.25	10	126880.3711	215	13.65
11	4574.241638	162	17.24	11	47948.55881	167	13.85
12	0	146	2.82	12	68316.29181	153	30.76
13	5838.443756	166	12.36	13	0	479	1.25
14	449.7214556	26	41.59	14	32992.91611	109	1.60
15	7472.707748	14	3.10	15	18782.56798	539	2.50
16	20002.53105	124	7.29	16	0	597	31.52
17	0	056	77.46	17	17914.39438	416	22.18
18	2919.049501	069	79.46	18	18311.41281	278	19.44
19	0	485	20.34	19	36226.25351	734	31.77
20	24352.15759	3041	11.37	20	48950.22202	415	29.55
21	0	2077	15.73	21	0	365	12.73
22	3175.864458	1234	6.88	22	0	937	43.27
23	6933.708191	201	41.53	23	22605.70526	628	21.28
24	4482.713699	261	28.23	24	33647.85004	1884	11.54
25	4170.306683	21	49.66	25	33567.1196	1049	24.23
26	5064.297199	219	44.17	26	25119.77386	973	38.18
27	3566.889524	247	3.71	27	0	1152	30.42
28	0	441	27.85	28	55128.37601	2821	1.78
				29	40356.28128	152	3.52
				30	64150.62714	197	2.16
