# Supplementary material for: Serological and molecular analysis of parvovirus B19 infection in Mayan women with systemic lupus erythematosus in Mexico
Source: Colomb Med (Cali). 2017 Sep 30;48(3):105–12. doi: 10.25100/cm.v48i3.2981 (PMC5687861; doi:10.25100/cm.v48i3.2981)
Supplement: Translation

# Translation: Análisis serológico y molecular de la infección por parvovirus B19 en mujeres mayas con lupus eritematoso sistémico en México

## Introducción

El lupus eritematoso sistémico (LES) es una enfermedad autoinmune inflamatoria sistémica crónica de etiología desconocida, causada por la interacción de factores genéticos y ambientales que contribuyen a la producción de autoanticuerpos frente a los auto-antígenos. La enfermedad tiene una distribución mundial y afecta principalmente a las mujeres ^1^^-^^3^. Países asiáticos como China, Hong Kong, Filipinas y Japón han reportado más casos, y otros como Estados Unidos, Francia, España, Reino Unido y algunas regiones de Australia, han presentado un aumento en los pacientes ^4^^,^^5^. Varios estudios se han realizado en pacientes de diferentes poblaciones (asiática, europea, americana), pero pocos en la población mexicana.

México tiene una población mestiza mezclada con una carga genética de amerindios y españoles ^6^. Los datos de ancestría derivados del proyecto HapMap, que incluyó a los mexicanos, muestra que los mestizos de Yucatán son el único grupo étnico con ascendencia amerindia que está geográficamente distante de otros grupos amerindios ^7^. Por otro lado, los individuos mexicanos con LES parecen tener una enfermedad más severa que la europea, una menor edad de inicio y una mayor frecuencia de brotes de actividad de la enfermedad. También se ha reportado que la prevalencia de LES en Yucatán (0.7%) es ligeramente superior a la prevalencia nacional (0,6%) ^8^^,^^9^.

Factores ambientales como infecciones bacterianas, parasitarias, fúngicas y virales se han asociado con la patogénesis de la enfermedad en pacientes genéticamente predispuestos ^3^^,^^10^. Se ha reportado que varios virus y bacterias pueden producir superantígenos que, a través de mecanismos tales como el efecto adyuvante (bystander) y el mimetismo molecular, inducen la activación de linfocitos T y B autoreactivos. Las partículas virales en los linfocitos B infectados pueden conducir a la producción de autoanticuerpos y citoquinas como el IFN-α, contribuyendo al mecanismo autoinmune e inflamatorio. El virus de Epstein-Barr (EBV), el citomegalovirus (CMV), el virus T-linfotrópico humano 1 (HTLV-1) y el parvovirus B19 (PV-B19) se han relacionado con la patogénesis del LES ^11^^,^^12^.

El PV-B19 humano, identificado en 1975 por Yvonne Cossart y sus colaboradores ^13^, es un virus pequeño de ADN de una sola hebra (22-24 nm de diámetro) que causa una variedad de enfermedades en los seres humanos. Su cápside icosaédrica se compone de dos proteínas estructurales idénticas, VP1 (83 kDa) y VP2 (58 kDa), excepto por un fragmento adicional de 277 aminoácidos en el amino terminal de VP1. Esta única región VP1 es externa a la cápside, con muchos epítopos lineales y actividad de fosfolipasa A2 (PLA2), lo que causa citotoxicidad e infectividad. PV-B19 también tiene la proteína no estructural NS1 (77kDa) involucrada en su transcripción y traducción ^14^. Se han identificado tres variantes genotípicas de PV-B19: el genotipo 1 tiene una distribución mundial; el genotipo 2 se ha detectado en pacientes de varios países europeos, Estados Unidos y Brasil; el genotipo 3 es más frecuente en África y menos frecuente en otras áreas geográficas ^15^^-^^22^.

PV-B19 se transmite por vía aérea (secreciones respiratorias) de individuos infectados, o por la transmisión parenteral a través de transfusiones de sangre y productos sanguíneos ^23^^-^^25^. El virus se replica en los eritroblastos de la médula ósea, que expresan el grupo sanguíneo antígeno P o globoside (Gb4), la integrina alfa5beta1 y la proteína Ku80 ^26^. La replicación viral, que conduce a la viremia al día 6, parece ser importante en la mayoría de las manifestaciones clínicas. La mayoría de las infecciones son asintomáticas o tienen cuadros clínicos leves, pero cuando la infección se asocia con trastornos clínicos influenciados por la edad o estado inmunológico y hematológico, presenta una amplia variedad de manifestaciones clínicas que pueden confundirse con enfermedades autoinmunes sistémicas como la artritis reumatoide (AR), esclerosis sistémica progresiva, síndrome de Sjögren (SS), vasculitis o SLE ^27^^-^^29^. La infección por PV-B19 puede diagnosticarse erróneamente como LES dé aparición reciente, pero al mismo tiempo, ambas pueden ocurrir simultáneamente en algunos pacientes.

PV-B19 es un virus ubicuo, distribuido en todo el mundo, que puede infectar a cualquier grupo de edad. La infección primaria ocurre generalmente en la niñez y adolescencia. La seroprevalencia (presencia de IgG específica que denota la infección pasada) aumenta con la edad. En los países industrializados, se calcula entre el 2% y el 10% de los niños menores de 5 años que pueden aumentar hasta el 50% a la edad de 15 años, y en los adultos varía entre el 40% y el 70%. A la edad de 70, llega a ser de 80% a 100% ^30^^,^^31^. Japón y Alemania han reportado altas tasas de infección en mujeres embarazadas ^32^^,^^33^.

En México, existen pocos estudios clínicos y epidemiológicos de PV-B19. Tapia *et al*., determinaron IgM e IgG en 128 personas de grupos considerados de alto riesgo de infección por PV-B19, y personas sanas de todas las edades y ambos sexos, en el Hospital de Enfermedades Infecciosas del Centro Médico La Raza. Los resultados mostraron la presencia de infección especialmente en mujeres (63.2%) de 25-44 años (48.4%), con exantemas, aborto habitual y anemia en pacientes inmunocomprometidos o trastornos hematológicos. En 61 pacientes (47.6%), se encontraron anticuerpos IgG elevados, y sólo 4 de ellos tenían IgM3 ^4^. Vera *et al*. ^35^, realizaron un estudio prospectivo preliminar en 102 mujeres embarazadas en dos ciudades rurales de Yucatán, México, encontrando una seroprevalencia de 5.9% de IgM y 11.8% de IgG; confirmando la presencia de infección por PV-B19 en estas poblaciones.

Diversos estudios se han centrado en el diagnóstico de la infección por PV-B19, pero la relación de PV-B19 con LES establecido no se ha estudiado en la población maya de México. Nuestro objetivo fue realizar un análisis serológico y molecular preliminar de la infección por PV-B19 en mujeres de la población maya con LES establecido y mujeres sanas. IgM e IgG anti-PVB19, presencia de ADN viral y carga viral se evaluaron en ambos grupos.

## Materiales y Métodos

### Pacientes con LES

Sesenta y seis mujeres con LES de origen maya fueron reclutadas del área de consulta eterna de Reumatología del Hospital Regional Agustín O'Horán e ISSSTE, Yucatán. El diagnóstico se estableció según los criterios del Colegio Americano de Reumatología (ACR) ^36^, y la actividad de la enfermedad fue evaluada por el puntaje de SLEDAI ^37^. Las mujeres con LES reportaron tener diferentes tiempos con la enfermedad. Se estudiaron 66 mujeres sanas del mismo origen sin antecedentes de enfermedades autoinmunes o infecciosas, como controles, y ninguna de ellas estaba recibiendo algún tratamiento. Todas las mujeres incluidas dieron su consentimiento informado, según la Declaración de Helsinki. El estudio fue aprobado por el Comité de Ética en Investigación del Hospital Agustin O'Horán de Yucatán (CIE-008-1-11). Todas las mujeres dieron 10 mL de sangre venosa periférica (sin anticoagulante) en una toma para obtener suero.

### IgM e IgG anti-PV-B19

Se utilizaron dos kits ELISA comercialmente disponibles para la detección de IgM anti-B19 (EIA-3504) y IgG anti-B19 (EIA-3503) (DRG Instruments GmbH, Alemania) ^38^. Los pocillos de la placa de microtitulación, como fase sólida, se recubrieron con el antígeno recombinante de PV-B19 (proteína VP1). El suero diluido de pacientes y controles, y los controles del kit listos para usar, se pipetean en estos pocillos. Durante la incubación los anticuerpos específicos de PV-B19 del suero positivo y los controles se unen al antígeno inmovilizado. Posteriormente, se añade la anti-IgG humana o anti-IgM conjugada a peroxidasa de rábano picante (HRP). La reacción se visualiza añadiendo tetrametilbenzidina (TMB) que genera un color azul. La reacción enzimática se detiene mediante la adición de una solución de ácido sulfúrico (H_2_SO_4_), que desarrolla un color amarillo. La intensidad del color es proporcional a la concentración de anticuerpos. La lectura se realizó a una longitud de onda de 450 nm en un lector de ELISA (modelo BioTek(r) ELx800), y la concentración de anticuerpos se determinó mediante la siguiente fórmula:

***Concentración del anticuerpo = (Abs) (10) / CO***

Dónde:

Abs = absorbancia de la muestra

10 = constante para comparar las absorbancias (control de corte y muestras control)

CO = absorbancia media del control de corte

La concentración de anticuerpos se expresa en DU (unidades DRG, medida exclusiva del proveedor utilizada para medir parámetros de inmunoglobulinas), tomando como referencia la absorbancia del control de corte. Cada ensayo se realizó por duplicado usando los controles positivos, negativos y de corte, contenidos en el kit. Los resultados fueron interpretados como sigue: IgM positivo ≥11 DU, IgG positivo ≥12 DU, IgM negativo ≤ 9 DU, e IgG negativo ≤ 8,5 DU, respectivamente.

### Aislamiento del ADN

La extracción de ADN se realizó en sueros positivos para IgM e IgG de pacientes y controles mediante el método de fenol saturado ^39^. Este procedimiento se basa en el método clásico de extracción de fenol / cloroformo usando 200 µL de muestras de suero. Se añadió solución de cloroformo-alcohol isoamílico (24:1) para separar proteínas, y el ADN se precipitó con etanol al 100% y acetato de amonio 7.5 M durante 24 h a -20º C. El ADN precipitado se lavó dos veces con etanol al 70%, se secó en el horno a 37° C durante 1 hora y después se resuspendió en 30 μL de agua ultrapura. Después de la incubación de 20 minutos a 56° C, el ADN se cuantificó en un espectrofotómetro (Nanodrop (tm) Thermo Scientific(r) 2000c), a longitudes de onda de 260 y 280 nm. El ADN se almacenó a -20º C hasta su uso.

### Clonación

Para determinar la presencia de ADN viral y cuantificar la carga viral, se clonó un segmento de la proteína NS1 a partir del ADN viral extraído del suero de un paciente diagnosticado con PV-B19 y se usó como control positivo. La banda de 168 pb correspondiente a la proteína NS1 se amplificó usando la Taq ADN polimerasa Maxima Hot Start (Thermo Scientific) y los cebadores (0.5 μM) como sigue: una desnaturalización inicial de 4 min a 95º C, seguida de 40 ciclos de desnaturalización (95º C durante 30 s), alineación del cebador (55º C, 30 s), etapa de extensión de 1 minuto a 72º C y una etapa final de in cubación a 72º C durante 10 min. Los productos de PCR se identificaron por electroforesis en agarosa al 1% teñida con GelRed (GelRedTM Nucleic Acid Gel Stain, Biotium). La banda se purificó mediante centrifugación (kit Wizard SV Gel y PCR Clean-up System, Promega), y se ligó al vector de clonación pCR 2.1-TOPO (kit de clonación TOPO-TA, Invitrogen por Life Technologies). El producto de ligación se introdujo en células de *E. coli* químicamente competentes (One Shot(r) TOP10 Competent Cells, Invitrogen por Life Technologies) a 42° C / 30 s, y se sembraron en medio sólido LB con kanamicina (50 μg / mL) durante la noche a 37° C. Se seleccionaron colonias blancas que expresaron el plásmido y se sembraron en el medio LB líquido durante la noche a 37° C. El plásmido se purificó (kit PureLink Quick Plasmid DNA Miniprep, Invitrogen por Life Technologies) y se visualizó mediante electroforesis en agarosa al 1% GelRed. La presencia del inserto se determinó mediante PCR usando la Taq ADN polimerasa Maxima Hot Start (Thermo Scientific). Los plásmidos purificados se analizaron mediante BLAST y se compararon con las secuencias reportadas del gen NS1 de PV-B19 en el GenBank.

### Detección del ADN de PV-B19

El ADN viral (15 ng) se amplificó mediante PCR en tiempo real, diseñado de acuerdo con la guía del MIQE ^40^. Los cebadores y sondas usados ​​en las reacciones de amplificación fueron descritos por Bonvicini *et al*. ^41^, (B19 cebador delantero 5'-CGCCTGGAACASTGAAACCC-3 ', B19 cebador inverso 5'-TCAACCCCWACTAACAGTTC-3', y sonda del genotipo 1 6FAMGTTGTAGCTGCATCGTGGGAAGAMGBNFQ). Se diseñaron por Applied Biosystems y se dirigieron contra la proteína no estructural 1 (NS1, 616-2631 nucleótidos) del genotipo 1 de PV-B19. Las condiciones de amplificación fueron las siguientes: 20 segundos de desnaturalización inicial a 95º C, seguido de 50 ciclos de amplificación: desnaturalización (95° C durante 3 s), alineación del cebador (55° C, 40 s), y una incubación final de 30 s a 60º C. La amplificación se llevó a cabo en el equipo de PCR en tiempo real StepOne (tm) utilizando TaqMan Fast Virus 1 step Master Mix (Applied Biosystems). El análisis del ADN viral (presencia / ausencia de NS1 y genotipo 1) se realizó de acuerdo con el CT (umbral de ciclo). El CT es el ciclo en el que el nivel de fluorescencia alcanza una cierta cantidad (el umbral). Este método utiliza directamente la información generada de CT para calcular la expresión relativa en las muestras blanco y de referencia, utilizando como referencia una muestra negativa ^42^.

### Carga viral

Las muestras positivas de IgM e IgG se cuantificaron mediante PCR en tiempo real utilizando los mismos cebadores y sonda, descritos anteriormente. La carga viral se cuantificó empleando un ensayo de curva estándar con diferentes números de copias del plásmido conteniendo el fragmento de 168 pb del gen NS1 (4,099 pb). El número de copias de la curva estándar de 6 puntos se determinó usando el software URI Genomics & Sequencing Center (http://cels.uri.edu/gsc/cndna.html). Este cálculo se basó en el supuesto de que el peso promedio de un par de bases (pb) es de 650 Daltons. Esto significa que un mol de un pb pesa 650 g y que el peso molecular de cualquier templete de ADN de doble hebra puede estimarse tomando el producto de su longitud (en pb) y 650. El inverso del peso molecular es el número de moles del templete presente en un gramo de material. Utilizando el número de Avogadro, 6.022x10^23^ moléculas / mol, se calcula el número de moléculas del templete por gramo. El número de copias del templete se estimó multiplicando por 1x10^9^ para convertir a ng y luego multiplicando por la cantidad de templete (en ng). La fórmula utilizada, a partir de una concentración inicial de 1ng, fue:

(1 ng x 6.022 x 10^23)^ / (4,099 x 1 x 10^9^ x 650) = 2.25 x 10^8^ copias. Se incluyeron cinco diluciones seriadas a la 10 (Tabla 1). Cada muestra y curva estándar se realizaron por triplicado. Las condiciones de amplificación fueron las siguientes: 2 min de precalentamiento a 50º C, 10 min de activación de polimerasa a 95º C, 50 ciclos de desnaturalización (95º C durante 15 s), alineación del cebador (55º C, 40 s), etapa de extensión 20 s a 72º C, y una etapa final de incubación a 60º C durante 30 s. La reacción de amplificación se realizó en el equipo de PCR en tiempo real StepOne (tm) (Applied Biosystems), utilizando la mezcla de reacción Maxima Probe / ROX (Thermo Scientific), primers (0.5 μM) y sonda (0.1 μM). El número de copias en las muestras se calculó con el software StepOne teniendo en cuenta el promedio de los valores de TC obtenidos con respecto a la curva estándar. La carga viral se expresa en copias por mililitro de suero (cps/mL).

**Tabla 1:** Número de copias de la curva estándar, determinada por triplicado con el software URI Genomics & Sequencing Center, como se describe en Material y métodos.

Curva estándar (ng)	Número de copias
1	2.25 x 10^8^
0.1	2.25 x 10^7^
0.01	2.25 x 10^6^
0.001	2.25 x 10^5^
0.0001	2.25 x 10^4^
0.00001	2.25 x 10^3^

### Análisis estadístico

Para evaluar la significancia de cualquier diferencia en los valores de IgM e IgG, y la carga viral (cps/mL) entre los pacientes con LES y sujetos control (*p* <0.05), se utilizó la prueba de rangos pareada de Wilcoxon. El análisis de correlación se realizó utilizando el coeficiente de correlación de Pearson. En todas las comparaciones, el nivel de significancia fue *p* <0.05, utilizando el programa Graph Pad Prism 5.

## Resultados

### Características de los pacientes con LES y de los controles

La edad promedio de los pacientes y controles fue de 39.03 y 38.18 años, respectivamente. El tiempo promedio con la enfermedad en pacientes fue de 9 años (Tabla 2). Todas estaban bajo tratamiento, 56.1% de ellas tenían enfermedad activa determinada por SLEDAI (>4).

**Tabla 2:** Características de los pacientes con LES (n = 66).

Características	Pacientes con LES
Edad promedio (años)	39.0
Promedio de duración con la enfermedad (años)	9.9
SLEDAI	%
Activos (≥ 4)	56.1
Inactivos (< 4)	43.9
Localidades en el Estado de Yucatán	%
Mérida	53.0
Hunucmá	3.0
Maxcanú	3.0
Motul	3.0
Peto	4.6
Progreso	3.0
Other in Yucatan State	30.3
Tratamiento	%
Prednisona	54.5*
Azathioprina	39.4*
Metotrexate	13.6*
Deflazacort	18.2*
Hydroxycloroquina	19.7*

SLEDAI: Índice de actividad de la enfermedad del lupus eritematoso sistémico.

* Porcentaje de pacientes que recibieron el fármaco en combinación con otro

### Niveles de IgM e IgG anti PV-B19

Se detectaron anticuerpos en el 42.4% (28/66) de los pacientes con LES y en el 53.0% (35/66) de los controles. Encontramos 14.3% (4/28) de los pacientes y 11.4% (4/35) de los controles sin niveles significativos de IgM (*p*= 0.7922). Por otro lado, 82.1% (23/28) de los pacientes y 82.9% (29/35) de los controles mostraron IgG, pero se detectaron niveles significativamente más altos en los pacientes (*p*= 0.0353) (Tabla 3 y Fig. 1). Sólo un paciente y dos controles mostraron IgG e IgM. Se encontró que el 58.3% (14/24) de los pacientes con una duración de enfermedad de 4 años o más presentó IgG, pero no se observó correlación. La correlación tampoco se observó en aquellos que presentaron menos de 4 años (41.7%, 10/24) (Fig. 2). Se realizó un análisis de asociación de IgG e IgM con el SLEDAI, pero no se observó correlación entre los niveles de IgM y la actividad de la enfermedad (SLEDAI >4). Sin embargo, los niveles de IgG mostraron correlación negativa significativa en pacientes con menor actividad de la enfermedad (SLEDAI <4) (Fig. 3).

**Tabla 3:** IgM e IgG anti-PV-B19 en pacientes con LES (n = 66) y controles (n = 66), analizados por ELISA como se describe en Material y métodos.

Anticuerpo	Pacientes con LES (%)	Controles (%)
IgM > 11 DU	4 (14.3)	4 (11.4)
IgG > 12 DU	23 (82.1)	29 (82.9)
IgG and IgM	1 (3.6)	2 (5.7)
Total	28 (42.4)	35 (53.0)

**Figura 1:** Niveles de IgM e IgG anti-PV-B19 en pacientes con LES (n= 28) y controles (n= 30), analizados por ELISA como se describe en Material y métodos. Los resultados expresados ​​en unidades DU se presentan en graficas de puntos con la media y SME (media del error estándar). Prueba de rangos pareada de Wilcoxon se utilizó para evaluar la diferencia de expresión entre los pacientes con LES y sujetos control (*p* <0.05).

**Figura 2:** Análisis de correlación de IgG anti-PV-B19 con el tiempo de evolución (≥4, o ≤4 años) en pacientes con LES. Los resultados se presentan en graficas de puntos. Se utilizó la prueba de correlación de Pearson para evaluar la correlación. r = coeficiente de correlación de Pearson; *p* < 0.05.

**Figura 3:** Análisis de correlación de IgM e IgG anti-PV-B19 con actividad de la enfermedad (SLEDAI >4 o <4) en pacientes con LES. Los resultados se presentan en graficas de puntos. Se utilizó la prueba de correlación de Pearson para evaluar la correlación. r= coeficiente de correlación de Pearson; *p* < 0.05.

### Detección de ADN PV-B19 y carga viral

El fragmento secuenciado de la proteína NS1, utilizado como control positivo, se analizó mediante BLAST y mostró 100% de homología con secuencias reportadas del gen NS1 de PV-B19 en el GenBank ^43^^-^^45^ (Figura S1). La secuencia se registró en el GenBank (BankIt1994458 Human KY680313). El ADN de PV-B19 del genotipo 1 se detectó en 86.4% (57/66) y 81.8% (54/66) de los pacientes y controles, respectivamente (Tabla S1). La carga viral se cuantificó en 28/66 pacientes con LES y 30/66 controles sanos, que fueron positivos para IgM e IgG (Tabla S2). Se encontró que el 67.9% (19/28) de los pacientes presentó carga viral: 10.7% (3/28) con IgM y 57.1% (16/28) con IgG. También se encontró carga viral en el 80,0% (24/30) de los controles: 13.3% (4/30) con IgM y 66.7% (20/30) con IgG, respectivamente (Tabla 4). La carga viral no se detectó en pacientes con IgM (1/28), IgG (7/28), o ambos (1/28), ni en controles con IgG (5/30), o IgM e IgG (1/30) (Tabla S2). No se encontró correlación de IgM o IgG con carga viral en ambos grupos; sin embargo, la carga viral fue significativamente mayor en los controles con IgG (Fig. 4). Se muestra un gráfico que representa el número de copias de la curva estándar con los valores de CT de una muestra (Fig. 5).

**Tabla 4:** Carga viral sérica (cps/mL) en pacientes con LES (n= 28) y controles (n= 30) con IgM o IgG, analizados por qPCR y ELISA, respectivamente, tal como se describe en Material y Métodos.

Carga viral/anticuerpos	Pacientes con SLE(%)	Controles (%)
cps/mL (+) IgM (+)	3/28 (10.7)	4/30 (13.3)
cps/mL (+) IgG (+)	16/28 (57.1)	20/30 (66.7)
cps/mL (-) IgM (+)	1/28 (3.6)	--
cps/mL (-) IgG (+)	7/28 (3)	5/30 (16.7)
cps/mL (-) IgM/IgG (+)	1/28 (3.6)	1/30 (3.3)

**Figura 4:** Carga viral en suero de pacientes con LES (28/66) y controles (31/66) con IgM o IgG, analizada por qPCR como se describe en Material y métodos. Los resultados expresados ​​en copias / mL se presentan en graficas de puntos con la media y SME (media del error estándar). Prueba de rangos pareada de Wilcoxon se utilizó para evaluar la diferencia de expresión entre los pacientes con LES y sujetos control (*p* <0.05).

**Figura 5:** Gráfico que representa el número de copias de la curva estándar con los valores de CT de una muestra analizada, tal como se describe en Material y métodos. Los símbolos rojos indican los puntos de la curva estándar y los azules de una muestra, tomados del software StepOne.

## Discusión

Durante la infección viral la respuesta inmune humoral es crucial para limitar la infección. En individuos inmunocompetentes, la viremia comienza 6 días después de la infección y disminuye días más tarde con la presencia de anticuerpos contra las proteínas VP1 y VP2. En la infección aguda, los anticuerpos IgM son detectables en los primeros 3 días de la infección e indetectables entre 60 y 90 días, pero pueden permanecer elevados entre 3 y 6 meses ^46^^-^^48^. Algunos autores reportan que la infección aguda por PV-B19 puede desencadenar su aparición o exacerbar el SLE preexistente ^49^^,^^50^.

Este es el primer informe sobre la infección por PV-B19 en mujeres con LES establecido de la población maya en México. Anticuerpos IgM contra VP1 se detectaron en pacientes con LES confirmado quienes mostraron actividad de la enfermedad (SLEDAI >5), y tenía un promedio de 9.8 años con la enfermedad. Aunque los niveles de IgM en los pacientes no fueron diferentes de los controles y no mostraron correlación con la actividad de la enfermedad, probablemente debido a la duración de la enfermedad y el tratamiento, los datos sugieren una infección reciente en nuestros pacientes y parece correlacionar con la reactivación de la enfermedad. Esto apoya que la infección por PV-B19 se asocia con el LES establecido como factor etiopatogénico, y correlaciona con lo sugerido por Ramos *et al*^51^.

La IgG se detecta días después de la IgM, indicando resolución de la infección e infección pasada o crónica, proporcionando inmunidad a lo largo de toda la vida ^47^^,^^48^. En nuestro estudio, se detectaron altos niveles de anticuerpos IgG en pacientes con LES, apoyando la infección pasada o crónica. A diferencia de los datos reportados por Pugliese *et al*. ^52^, que encontraron una correlación significativa entre IgG anti-PV-B19 y SLE, no encontramos correlación entre IgG y SLEDAI; la correlación inversa observada entre IgG con baja actividad de la enfermedad (SLEDAI <4), se debe probablemente a los altos valores de IgG en un solo paciente, que consideramos no representativo. Nuestros datos sugieren que los niveles de IgG parecen aumentar en los pacientes con LES probablemente debido a la terapia que apoyo la infección crónica de PV-B19, ya que todos nuestros pacientes estaban siendo tratados con antiinflamatorios, corticosteroides y agentes inmunosupresores. A este respecto, se ha descrito que el uso de corticosteroides, agentes inmunosupresores y terapias biológicas puede aumentar el riesgo de infección viral en pacientes con LES y la infección por PV-B19 puede convertirse en crónica o grave en ellos ^53^^,^^54^. Sin embargo, estudios longitudinales son necesarios para confirmar esto.

Se detectó el genotipo 1 de PV-B19 en pacientes con SLE y controles, y se cuantificó la carga viral en aquellos pacientes y controles con altos niveles de anticuerpos IgM o IgG. No se encontró correlación entre los anticuerpos IgM o IgG y la carga viral en ambos grupos; sin embargo, se encontró una mayor carga viral en los controles confirmando la presencia de PV-B19 en la región, y apoyando la prevalencia de infección en la población maya. Los anticuerpos IgG y la carga viral en pacientes parecen apoyar la infección crónica asociada con la inmunosupresión mediada por la terapia. La carga viral no se detectó en algunos pacientes con IgM, IgG o ambos. En este sentido, se ha informado que la viremia desaparece al día 10 después de la infección, mientras que la IgM (10-12 días) e IgG (14 días) comienzan a sintetizarse. En esta etapa, no se detectan partículas virales, indicando que la razón para no detectar el ADN viral en algunos de nuestros pacientes y controles con alto título de IgG y / o IgM, podría ser que estos estaban en el día 12 de la infección, cuando el virus no está presente. Sin embargo, estudios longitudinales son necesarios para confirmar esto.

En nuestras mujeres control, no se observaron síntomas clínicos sugestivos de infección reciente o enfermedad, pero se detectaron anticuerpos IgM e IgG, así como presencia de ADN y carga viral. A pesar de las diferencias en el tamaño de muestra y las poblaciones estudiadas, los datos apoyan la seroprevalencia de IgM e IgG encontrada por Vera *et al*. ^35^, y confirma la circulación del virus en la población maya. La carga viral no se detectó en algunos controles con anticuerpos IgG o IgM / IgG. A este respecto, se ha reportado que los individuos inmunocompetentes producen anticuerpos que eliminan efectivamente la viremia a los pocos días de la infección, y la infección no suele desarrollarse, es asintomática o tiene manifestaciones clínicas leves (como el resfriado común) ^55^, lo que parece correlacionar con lo que encontramos en nuestros controles. Por otra parte, también se han registrado casos raros de infección crónica por PV-B19 en individuos sanos, con afectación del sistema nervioso central, causando síntomas inespecíficos como fatiga, fiebre, artralgia y mialgia, lo que puede dificultar el diagnóstico ^56^. Ninguna de nuestras mujeres control manifestó algunos de estos síntomas, sin embargo, los estudios longitudinales son necesarios para evaluar la asociación de la infección de PV-B19 con trastornos neurológicos, autoinmunes o hematológicos en la población inmunocompetente maya.

## Conclusión

La alta prevalencia de PV-B19 en Yucatán y la presencia de IgM, IgG y carga viral en mujeres mayas con LES establecido sugieren que la infección por PV-B19 podría ser un factor ambiental para desencadenar o reactivar el LES. Sin embargo, estudios longitudinales y una muestra mayor son necesarios para confirmar la asociación de PV-B19 con el desarrollo de LES, así como el efecto de la terapia inmunosupresora sobre el resurgimiento del virus.

## Figures and Tables

**Figura S1:** Alineamiento del fragment de la secuencia de la proteína NS1 de PV-B19 con la secuencia reportada en el GenBank.

## Anexos

**Tabla S1:** Presencia/ausencia de DNA viral e IgM o IgG en patientes SLE (n= 66) y controles (n= 66), analizados por PCR y ELISA, respectivamente, como se describio en Materiales y metodos.

Pacientes con LES	Presencia Ausencia	IgM (DU)	IgG (DU)	Sujetos sanos	Presencia Ausencia	IgM (DU)	IgG (DU)
1	P	2.50	3.74	1	A	2.40	2.20
2	P	1.90	1.60	2	P	2.80	1.76
3	P	3.83	90.75	3	A	2.00	4.80
4	P	5.95	5.60	4	P	2.80	1.40
5	P	4.05	1.83	5	P	3.70	4.10
6	P	4.42	16.48	6	P	6.70	1.70
7	A	5.20	3.00	7	P	2.60	2.42
8	P	2.46	16.63	8	P	3.30	7.60
9	P	2.57	34.12	9	A	1.40	6.10
10	P	6.99	70.16	10	P	3.11	3.53
11	P	4.75	88.25	11	P	2.00	2.10
12	P	1.62	17.24	12	P	2.28	14.54
13	A	2.10	3.90	13	P	5.70	2.00
14	P	1.46	2.82	14	P	1.82	7.49
15	A	0.00	1.53	15	P	2.78	7.46
16	A	1.44	2.83	16	P	4.24	1.39
17	A	1.38	9.98	17	P	4.20	2.59
18	P	7.13	2.88	18	P	1.64	1.16
19	P	2.19	3.37	19	A	2.00	4.20
20	P	1.66	12.36	20	P	3.69	41.26
21	P	2.60	41.59	21	P	2.23	86.02
22	A	4.10	1.70	22	A	2.04	2.71
23	P	1.40	2.00	23	P	3.13	24.94
24	P	1.40	3.10	24	P	3.64	13.72
25	P	4.10	2.50	25	P	2.06	22.42
26	P	7.10	1.10	26	P	2.48	4.21
27	A	5.38	3.10	27	P	2.15	13.65
28	P	2.00	1.40	28	A	6.90	6.60
29	P	1.24	7.29	29	P	1.80	17.44
30	P	1.11	2.40	30	A	1.70	3.08
31	P	5.15	2.73	31	P	2.18	2.84
32	P	5.86	2.76	32	P	1.67	13.85
33	P	5.08	2.29	33	P	2.50	7.50
34	P	2.81	3.41	34	P	2.23	2.26
35	P	0.60	2.00	35	P	1.53	30.76
36	P	0.66	0.79	36	A	2.65	1.95
37	P	5.72	1.20	37	P	4.79	1.25
38	P	3.73	1.75	38	P	1.09	1.60
39	P	0.56	77.46	39	P	5.39	2.50
40	P	0.69	79.46	40	P	3.70	2.80
41	P	5.12	8.31	41	P	1.51	1.60
42	P	4.85	20.34	42	A	1.76	0.00
43	P	6.62	6.98	43	A	5.10	2.60
44	P	30.41	11.37	44	P	5.97	31.52
45	P	20.77	15.73	45	P	4.16	22.18
46	P	8.75	11.15	46	P	2.70	5.40
47	P	12.34	6.88	47	P	14.42	4.21
48	P	5.77	1.02	48	P	2.78	19.44
49	P	2.01	41.53	49	P	7.34	31.77
50	P	4.32	7.35	50	P	4.15	29.55
51	P	2.43	7.37	51	P	1.80	8.27
52	A	11.02	11.34	52	P	4.15	11.43
53	A	1.96	3.35	53	P	3.65	12.73
54	P	4.16	9.51	54	P	2.92	20.17
55	P	4.89	4.65	55	P	9.37	43.27
56	P	1.73	7.91	56	-	3.10	11.81
57	P	2.61	28.23	57	P	6.28	21.28
58	P	1.37	4.00	58	P	18.84	11.54
59	P	2.62	0.00	59	P	10.49	24.23
60	P	1.61	5.13	60	P	9.73	38.18
61	P	2.52	10.54	61	P	11.52	30.42
62	P	2.08	9.18	62	P	14.41	16.08
63	P	2.1.	49.66	63	P	28.21	1.78
64	P	2.19	44.17	64	P	1.52	3.52
65	P	2.47	3.71	65	P	1.97	2.16
66	P	4.41	27.85	66	-	8.19	9.43

Presencia: P

Ausencia: A

**Tabla S2:** Carga viral en suero (cps/mL) en patientes SLE y controles con IgM o IgG, analizados por qPCR y ELISA, respectivamente, como se describio en Materiales y métodos.

Pacientes con LES	cps/mL	IgM (DU)	IgG (DU)	Sujetos sanos	cps/mL	IgM (DU)	IgG (DU)
1	4201.636791	25	3.74	1	159046.3715	28	1.76
2	4117.944717	19	1.60	2	68096.33636	26	2.42
3	0	383	90.75	3	0	311	3.53
4	9168.199539	595	5.60	4	122609.1675	228	14.54
5	0	405	1.83	5	5914.156437	42	2.59
6	0	442	16.48	6	67065.8493	223	86.02
7	26209.05876	246	16.63	7	16777.81105	313	24.94
8	0	257	34.12	8	94853.63007	364	13.72
9	8431.143761	699	70.16	9	39084.53369	206	22.42
10	2591.694593	475	88.25	10	126880.3711	215	13.65
11	4574.241638	162	17.24	11	47948.55881	167	13.85
12	0	146	2.82	12	68316.29181	153	30.76
13	5838.443756	166	12.36	13	0	479	1.25
14	449.7214556	26	41.59	14	32992.91611	109	1.60
15	7472.707748	14	3.10	15	18782.56798	539	2.50
16	20002.53105	124	7.29	16	0	597	31.52
17	0	056	77.46	17	17914.39438	416	22.18
18	2919.049501	069	79.46	18	18311.41281	278	19.44
19	0	485	20.34	19	36226.25351	734	31.77
20	24352.15759	3041	11.37	20	48950.22202	415	29.55
21	0	2077	15.73	21	0	365	12.73
22	3175.864458	1234	6.88	22	0	937	43.27
23	6933.708191	201	41.53	23	22605.70526	628	21.28
24	4482.713699	261	28.23	24	33647.85004	1884	11.54
25	4170.306683	21	49.66	25	33567.1196	1049	24.23
26	5064.297199	219	44.17	26	25119.77386	973	38.18
27	3566.889524	247	3.71	27	0	1152	30.42
28	0	441	27.85	28	55128.37601	2821	1.78
				29	40356.28128	152	3.52
				30	64150.62714	197	2.16
